# Rheumatoid Neuropathy: A Brief Overview

**DOI:** 10.7759/cureus.34127

**Published:** 2023-01-24

**Authors:** Bhavya Rajeshwari, Sunil Kumar

**Affiliations:** 1 Department of Medicine, Jawaharlal Nehru Medical College, Datta Meghe Institute of Higher Education and Research, Wardha, IND

**Keywords:** nerve conduction study, carpel tunnel syndrome, mononeuropathies, peripheral neuropathy, rheumatoid arthritis

## Abstract

Rheumatoid arthritis is an autoimmune disease commonly found in humans. It is characterized by stiffness and swelling of the joints, along with fatigue and malaise. Rheumatoid neuropathy is a neuropathy that arises as a complication of rheumatoid arthritis. The primary objective of this review article is to provide a detailed account of the various aspects of this neurological complication ranging from its incidence, clinical features, and diagnosis. After searching through various published review articles and textbooks, rheumatoid neuropathy is one of the most common complications of rheumatoid arthritis. Out of all types of neuropathies. the most observed is entrapment neuropathy. Carpel tunnel syndrome is the most common type of entrapment neuropathy. There seems to be a greater predilection of rheumatoid neuropathy in females compared to males. A direct relation exists between rheumatoid factor and the occurrence of neuropathy. Some clinical features of rheumatoid neuropathy include stiffness in hands and feet, burning and tingling, stabbing pain, occasional weakness, and numbness in several cases. The common modalities of diagnosis are history, clinical examination, blood test, magnetic resonance imaging, nerve conduction study, and tissue biopsy. From the above-mentioned modalities, nerve conduction studies must be chosen as they can detect latent cases quickly and effective treatment can be initiated immediately. Finally, we outline the treatment plan for the disease which can be divided into medical and surgical management. Medical management consists of symptomatic treatment such as analgesics, anticonvulsants, and antidepressants, while surgical management is the last resort and consists of nerve compression.

## Introduction and background

Rheumatoid arthritis is a chronic inflammatory autoimmune condition of an uncertain cause in which symmetric polyarthritis is seen [1]. It mainly affects small and large joints [2]. The onset of rheumatoid arthritis can be at any age, mainly at 40-60 years of age [3]. It manifests in various extra-articular ways, including peripheral neuropathy, interstitial lung disease, and ocular conditions, such as episcleritis, scleritis, and vasculitis [2,4]. The chance of developing extra-articular manifestations occurs with inadequately treated rheumatoid arthritis [3]. Patients with rheumatoid arthritis have an approximately 50% chance of developing peripheral neuropathy [1]. Peripheral neuropathy, which typically results in weakness, numbness, and discomfort in the hands and feet, is caused by injury to the nerves external to the brain and spinal cord [5]. It is called rheumatoid neuropathy because these occur as secondary manifestations due to rheumatoid arthritis. It may lead to disability in the patient. Although peripheral neuropathy is not an individual marker of mortality, peripheral nervous system involvement is frequently an early indicator of the generalization of inflammatory illness [6]. Entrapment, vasculitis-related nerve ischemia, or medications used to treat the illness, such as tumor necrosis blockers and leflunomide, are the fundamental causes of rheumatoid neuropathy [1].

## Review

Methodology

**Figure 1:**
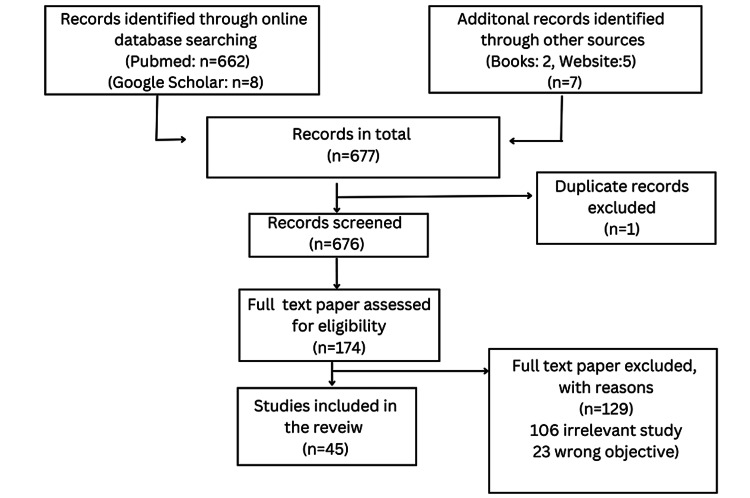
Flowchart showing the selection of included research studies. Figure credits: Bhavya Rajeshwari and Sunil Kumar.

This review article focuses on describing the incidence, clinical features, diagnostic modalities, and treatment of rheumatoid neuropathy which is an often-seen complication of rheumatoid arthritis. A thorough and comprehensive analysis of the research topic was performed using online databases such as PubMed and Google Scholar. The search strategy was tailored to individual databases and the following keywords were used: Rheumatoid Neuropathy, Rheumatoid arthritis, and Peripheral Neuropathy. The research criteria had no restriction on the publication date. Clinical studies (observational and trials) and review articles on rheumatoid neuropathy were included in this article. Studies on drug trials and neuropathy caused by other autoimmune diseases such as Sjögren syndrome were excluded. In total, 45 references were included in this article, of which five are websites and two are medicine textbooks. The selection of the included studies is depicted in Figure 1.

Incidence

According to reports, it affects between 0.1% and 2.0% of people globally [7]. The occurrence of rheumatoid arthritis is thought to be influenced by a wide range of genetic and environmental variables both internationally and locally [8,9]. Based on self-reported data from the 2014-2015 National Health Survey (NHS), Australia recorded the highest incidence of rheumatoid arthritis (2%) in the world at the population level due to the high prevalence of the gene (such as *PTPN22 *risk allele) predisposing to the disease [7,10]. The research found the most significant documented rheumatoid arthritis prevalence at the community level among American Indigenous groups. In contrast, rural populations in South Africa and Nigeria have shown lower incidences or no rheumatoid arthritis [7]. The occurrence of rheumatoid neuropathy out of all cases is approximately four to five people in 1,000 [11]. Rheumatoid neuropathy is one of the most common causes of morbidity in a patient with rheumatoid arthritis [12]. Specific studies were conducted to show the relationship between neuropathy and rheumatoid arthritis. These studies also help to gain an idea about which kind of neuropathies are more common and what age group and gender are the most affected.

The first case series of patients with rheumatoid arthritis who also had nerve damage was published by Hart and Goldin. They conducted the study among 42 patients, of whom 18 were men and 24 were women in the age group of 36 to 88 years. They noted that men showed high occurrence, and the onset was abrupt in 13 cases, while gradual and insidious in the rest. They also stated that, primarily, the sensory loss was seen. Out of all 42 cases, 18 showed motor weakness, and the absence of ankle reflex was also noted, which was bilateral in eight cases and unilateral in four. They also concluded that neuropathy mainly occurs in patients with rheumatic nodules and advanced rheumatic disease [13]. Aneja et al. conducted a study among 100 rheumatoid arthritis patients to assess the pattern and frequency of neuropathy. Out of the 100 patients, 66 were screened, with a mean age of 42 years. On the neurological examination of these 66 cases, it was found that 16 had both sensory and motor abnormalities. Upon nerve conduction studies, 25 patients showed an abnormality, with the majority showing entrapment neuropathy, and none showing autonomic involvement. They concluded that there is a high frequency of subclinical neuropathy in patients from India [14]. Autonomic symptoms are not usually well recorded in nerve conduction studies due to the involvement of small fibers, certain studies can be employed. Some of these include quantitative sensory testing (QST) along with skin biopsy, as was demonstrated by Fabry et al. The objective for performing QST was to determine the threshold for not just warm but also cold sensations. Skin biopsy coupled with quantifiable measurement of the intraepidermal nerve fibers (IENF) is also beneficial in the determination of small nerve fiber damage [15].

Biswas et al. conducted a study in a tertiary care hospital with 74 patients with rheumatoid arthritis. This study was conducted over two years, wherein the researchers found the occurrence of peripheral neuropathy both clinically and electrophysiologically. It was pointed out that the majority of cases were between 41 and 50 years of age. In a nerve conduction velocity study, they noted that out of all, 29 patients showed peripheral neuropathy, and, clinically, it was found that out of these 29 patients, 24 did not show any symptoms. Approximately 15 patients showed pure sensory involvement, whereas three patients showed carpel tunnel syndrome. The study also found that sensory involvement is more commonly seen than sensorimotor. Still, some other studies, such as Nadakar et al. and Hamed et al., indicated that sensorimotor is more widely seen. They also noticed the association of rheumatoid factor positivity with the occurrence of peripheral neuropathy. The primary flaw of the study was the absence of nerve biopsy tests, the pathology equivalent of a gold standard, to support their electrophysiological findings [16]. In the study by Bharadwaj et al. [17], neuropathy was seen in 12 rheumatoid arthritis patients out of 36. Not excluding patients with diabetes or drunkenness, they discussed leflunomide as a confounding factor. However, the drawback of this study was that the nerve conduction investigations were only performed among patients with clinical signs of neuropathy and were missing in subclinical cases [16].

Sim et al. conducted a study to assess the prevalence of rheumatoid arthritis and peripheral neuropathy. In their study, a total of 30 patients were studied, of whom approximately 10 were found to have peripheral neuropathy. They mentioned this being seen more commonly in patients above 60 years of age and there is a strong correlation between peripheral neuropathy and anti-cyclic citrullinated peptide antibody, with 80% of patients who have peripheral neuropathy also having the anti-cyclic citrullinated peptide antibody. The study concluded that neuropathic symptoms are common, and it is difficult to differentiate between the symptoms of rheumatoid arthritis and peripheral neuropathy [18]. Kealey et al. conducted a preliminary study with 89 patients with rheumatoid arthritis to assess the incidence of peripheral neuropathy. Their study involved a combination of old and new diagnoses that were taken into consideration. Approximately 50% of patients were asymptomatic and subclinical. The most common diagnosis was the loss of simple, delicate touch, followed by loss of ankle reflex. The study concluded that peripheral neuropathy is associated with increasing age, duration of disease, and increased disease severity in the geriatric population, which can lead to functional disabilities [2]. A study conducted by Mold et al. concluded that there exists a link between the frequency of peripheral neuropathy in the elderly population [19]. George et al. conducted a retrospective, observational, hospital-based study with 60 patients in a tertiary care hospital in eastern India. They included patients having peripheral neuropathy due to rheumatic arthritis, excluding other metabolic causes, such as diabetes and Sjogren’s syndrome. It was also noted that the mean age of onset was 44 years, and the maximum cases were seen in females. Of these, 30% of the patients were suffering from neuropathy, a few patients showed involvement of only the upper limb, a few showed involvement of only the lower limb, and a few showed involvement of both. They concluded that early diagnosis would help with early management and prevent the disease from further increasing and causing complications [12]. All inferences are summarized in Table 1.

**Table 1:** Various studies and their observations. Table credits: Bhavya Rajeshwari and Sunil Kumar.

Author	Title	Observation
Hart et al. [13]	Rheumatoid neuropathy	Higher occurrence of rheumatoid neuropathy in men than women. Sensory loss is seen more commonly than motor loss. In the motor loss, bilateral involvement is more common than unilateral involvement
Mold et al. [19]	The prevalence, predictors, and consequences of peripheral sensory neuropathy in older patients	Higher frequency of peripheral neuropathy in the older population
Bharadwaj et al. [17]	Interstitial lung disease and neuropathy as predominant extra-articular manifestations in patients with rheumatoid arthritis: a prospective study	Neuropathy was observed in one-third of the patients with rheumatoid arthritis.
Aneja et al. [14]	Prevalence of peripheral neuropathy in patients with newly diagnosed rheumatoid arthritis	Both sensory and motor involvement were observed. The most common type of neuropathy in patients was entrapment neuropathy
Biswas et al. [16]	Prevalence, types, clinical associations, and determinants of peripheral neuropathy in rheumatoid patients	The majority of patients were found to be asymptomatic. Sensory was more commonly seen than sensorimotor
Sim et al. [18]	Assessment of peripheral neuropathy in patients with rheumatoid arthritis who complain of neurologic symptoms	Rheumatoid neuropathy was more commonly seen in the elderly (60 years). A positive relationship between peripheral neuropathy and anti-cyclic citrullinated peptide antibody was observed
Kealey et al. [2]	Prevalence and patterns of peripheral neuropathy in patients of rheumatoid arthritis	Most patients were asymptomatic. The most common symptom was the loss of superficial delicate touch. Increased severity of disease was noted in the elderly population.
Fabry et al. [15]	Which method for diagnosing small fibre neuropathy	The most sensitive test for diagnosing small fiber neuropathy was quantitative sensory testing. The application of skin biopsy for reference in the diagnosis of small fiber neuropathy is yet to be established
Thomas et al. [12]	Proportion of peripheral neuropathy in newly diagnosed rheumatoid arthritis - a single centre retrospective observational study	Maximum incidence was seen in females. Involvement of both the upper and lower limb was observed

Types

**Figure 2:**
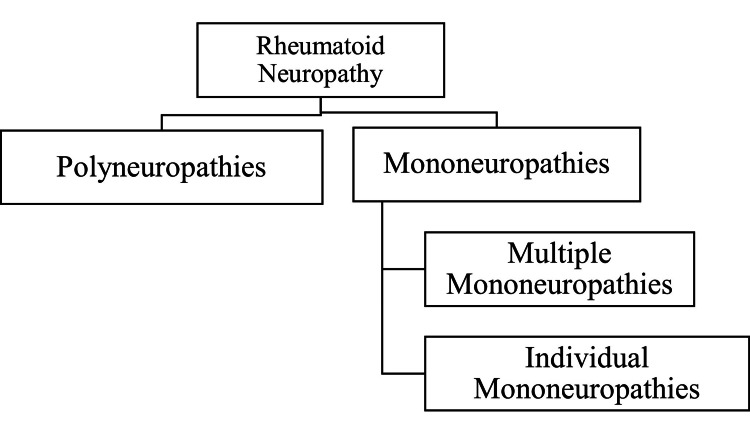
Flowchart showing the types of rheumatoid neuropathy. Figure credit: Bhavya Rajeshwari and Sunil Kumar.

As demonstrated in Figure 2, there are two types of neuropathies, namely, polyneuropathies and mononeuropathies. Mononeuropathies can be further divided into multiple and individual mononeuropathies.

Rheumatoid Arthritis Polyneuropathies

These can either be subclinical, pure sensory, or, less often, sensorimotor axonopathies [20]. One of the most reasons is vasculitis. Because the pathologic alterations start in the highly distal regions of the most extensive and longest nerves and move down from the afflicted fibers toward their nerve cell bodies, polyneuropathies are characterized, in particular, by a highly uniform spread of weakening that is distal (dying-back neuropathy or distal axonopathy) [21].

Rheumatoid Arthritis Multiple Mononeuropathies

These usually occur because of vasculitis, which occurs in a chronic case of rheumatoid arthritis [21]. Impairment to the vasa nervorum caused by inflammation leads to nerve ischemia that mimics polyarteritis nodosa-related vasculitis neuropathy [22]. Lesions of the nerve include enormous degeneration of the axons. There is a direct correlation between the extent of damage to the axons and the deficit of the sensorimotor system, along with the rate at which a patient can recover [23]. This implies that the lesser the damage to the axons, the lesser the sensorimotor loss and the faster the recovery rate.

Rheumatoid Arthritis Individual Mononeuropathies

These kinds of neuropathies mainly result due to entrapment (that is, compression) and is one of the most frequent demonstrations of the peripheral nervous system [22]. Some of the causes of compression include but are not limited to joint subluxation, synovitis, and tenosynovitis. About one-tenth of those with rheumatoid arthritis present with symptoms of carpel tunnel syndrome [22]. Entrapment neuropathies, which frequently affect the median, ulnar, radial, or anterior tibial nerve, develop due to proliferative synovitis or joint abnormalities [21]. Particular examples of this include carpal tunnel syndrome is usually seen more than other manifestations and might be considered one of the presenting signs [24]. Being a rheumatic patient increases the chances of acquiring it by two folds [20,25]. It comprises compression of the median nerve when it approaches the wrist. A patient suffering from carpel tunnel syndrome will complain of loss of sensation and numbness in the hands. This is more pronounced at night or while performing tasks that involve flexion of the wrist, such as driving or typing [22]. Tarsal tunnel syndrome is caused by compression of the distal tibial nerve [25]. It manifests as a loss of sensations coupled with pain over the plantar aspect of the affected foot. Pain is produced when we tap over the tarsal tunnel, known as the Tinel sign [22]. Some rarer forms of neuropathy include ulnar neuropathy at the elbow or wrist, posterior interosseous nerve syndrome, femoral neuropathy, and peroneal neuropathy [26].

Clinical features

Risk Factors

The incidence of acquiring rheumatoid neuropathy increases with age and in patients having rheumatoid arthritis for a long duration. Apart from these, some other risk factors include diabetes, increased intake of alcohol, and deficiency of vitamins (such as vitamin D). Chances are also increased if the family has a history of neuropathy [11].

Symptoms and Signs

Approximately 50% of patients are either asymptomatic or have subclinical neuropathy [11]. Therefore, on the basis of clinical manifestations, these cannot be described. Individuals present with pain, swelling, and stiffness in a symmetrical pattern, predominantly affecting the smaller joints of the hands and feet [3]. It might be challenging to tell the difference between peripheral neuropathy symptoms and arthritis symptoms because these symptoms coincide and imitate those of arthritis [21]. Rheumatoid arthritis symptoms include stiffness in the hands and feet that lasts longer than an hour in the morning. The proximal interphalangeal joints (PIPJs), metacarpophalangeal joints (MCPJs), wrists, elbows, and shoulders are individually examined. Classically, the distal interphalangeal joints (DIPJs) are spared in rheumatoid arthritis [3]. Clinical manifestations may be burning or tingling that can start gradually, going up from the feet or hands into the legs or arms. At times, it may even give a feeling of stabbing pain. The neuropathy in rheumatoid arthritis characteristically presents as a glove and stocking pattern [3]. There can also be urinary incontinence and syncope [27]. The specific symptoms depend on which nerves are affected, such as sensory nerves in the skin that is responsible for temperature, pain, or touch, and motor nerves that control muscle action [11]. Autonomic nerves are required to perform the body’s essential functions, such as blood pressure, heart rate, and digestion. In patients with rheumatoid arthritis, one of the more common findings is swelling and tenderness over multiple joints [3]. These include the absence of decreased deep and superficial reflexes such as ankle reflex, biceps reflex, and knee jerk [28].

Diagnosis 

History should be adequately taken by following proper procedure and inquiring about symptoms of neuropathy which are mentioned in the above section, followed by an inquiry about symptoms related to rheumatoid arthritis [11]. Clinical examination should be performed carefully as patients can be asymptomatic. Reflexes, coordination, balance, strength, and testing for the sensory system should be carried out [11]. A basic workup that needs to be performed includes a blood test which can be done to assess whether there are any other underlying causes, such as vitamin deficiency, electrolyte imbalance, or any comorbid condition, such as diabetes mellitus or rheumatoid arthritis [29,30]. An increase in blood platelet levels and C-reactive protein levels and a decrease in blood protein levels, mainly albumin, should also be assessed as these can act as risk factors for developing peripheral neuropathy. The rheumatoid factor should also be assessed as few past studies have shown that there can be a relation between rheumatoid factor and neural damage. The higher the value of rheumatoid factor the higher the chances of developing neuropathy. Detection of IgM rheumatoid factor in serum, as well as anti-CCP antibodies, is also beneficial in diagnosis [12]. Monoclonal gammopathies are frequently seen in patients with polyneuropathy. To identify the same serum protein immunofixation electrophoresis can be performed [31].

Disease activity score (DAS28) should also be checked, which helps to assess disease activity [12,32]. MRI should be done to see the extent of damage done to the nerve. Ultrasonography can be done for patients who are not able to afford MRI. Nerve conduction study and electromyography can be done to assess the activity of the nerve; they mainly asses large, myelinated nerve fibers [33]. Electromyography can be done in cases with diagnostic ambiguity, independent or multifocal processes, and helps in validating the probable diagnosis and ruling out widespread imitators such as mononeuropathies or radiculopathies [34]. Nerve conduction studies can be used for the evaluation of peripheral neuropathy in patients who are subclinical. Therefore, it is recommended to do this in all patients with rheumatoid arthritis [12,35]. A tissue biopsy to validate the diagnosis and a quantitative sudomotor axon reflex test, which examines a patient’s sweating capabilities, are less common testing modalities. Specifically, when talking about patients with sensory neuropathy, one should check for vasculitis, as it is one of the most common causes [36]. Vasculitis can be encountered by the presence of skin lesions along with nail fold infarcts and splinter hemorrhages [37].

Treatment

Conventional synthetic disease-modifying anti-rheumatic drugs (csDMARD) should be used to treat rheumatoid arthritis as soon as the diagnosis is confirmed [38]. Because methotrexate has the most clinical experience in monotherapy, it should be the top choice among csDMARDs for first-line therapy despite the lack of a controlled comparison between methotrexate and csDMARDs [39]. The standard starting dose of methotrexate is 15 mg/week, which can be gradually increased to 25 mg/week. It is advised to combine it with glucocorticoids [40]. The preferred mode of administration is subcutaneous as its bioavailability is reduced [34]. Patients with a higher baseline disease activity and rheumatoid factor-positive patients have an increased risk of methotrexate failure due to inefficacy [41]. If methotrexate cannot be used, for example, because of intolerance or contraindications, leflunomide (20 mg/week) or sulfasalazine (2 g/day) should be initiated [42].

Adjustment of therapy should be initiated in case of the following two situations: in the event of non-satisfactory response after 12 weeks of initiation of therapy, and in the absence of any improvement in the condition after 24 weeks of medication [39]. Categorizing patients using prognostic markers can be beneficial in ascertaining the ideal course of treatment for each individual. There is an inverse relationship between the predictive value of a marker and the course of the disease. The lower the prognostic value, the faster the disease progresses. Some prognostic markers include, but are not limited to, early damage of the joints, detection of autoantibodies, etc. In such a scenario, biological DMARD is recommended [38]. In the event of a combination of poor prognostic markers and a moderate grade of ailment, the application of one more csDMARD is suggested [39].

Infliximab, a chimeric IgGI monoclonal antibody derived from either humans or rodents, was the primary tumor necrosis factor-alpha inhibitor to gain approval for therapeutic purposes [43]. A standard dosage of 3-5 mg/kg via the intravenous route is advised. It should be given once every two months (approximately eight weeks) [44]. The principal objective of therapy is managing not just the disease but also its neuropathic complications. We can alleviate and stop the symptoms of peripheral neuropathy from occurring by prescribing DMARDs. Analgesics include non-steroidal anti-inflammatory drugs (paracetamol), anti-convulsants include gabapentin (Gralise, Neurontin, Horizon) and pregabalin (Lyrica), which have been helpful in symptomatic relief of nerve pain, topical treatments, antidepressants include certain tricyclic antidepressants, such as amitriptyline, doxepin, and nortriptyline (Pamelor), which are also helpful owing to their ability to interfere with the pain receptors present in the brain and spinal cord. Therapies and procedures include physiotherapy which has also proven to be beneficial in not just pain relief but also improvement in the range of movements. These procedures are practical when a patient complains of muscle weakness. Another device that is helpful in pain relief is the transcutaneous electrical nerve stimulation unit. This device is battery-operated, and the working mechanism is based on generating electrical impulses with a low voltage, which is then transmitted to the skin. The device usage duration is an average of half an hour daily for a month [45].

In the event of sensory neuropathy, which is in its very initial stages, the course of action comprises careful management at timely intervals. However, when sensory neuropathy advances to sensorimotor neuropathy, or in the likelihood of mononeuritis multiplex, a quick introduction of aggressive therapy coupled with invasive diagnostic procedures should be performed [36]. Future advancements will affect how rheumatoid arthritis patients with peripheral neuropathy are treated. As soon as following the possible diagnosis, we advise rheumatoid arthritis patients to have the afflicted nerve surgically decompressed. However, as more potent medications for rheumatoid arthritis are developed, conservative therapy may be preferred over surgery [45].

## Conclusions

In this review article, we have provided a brief overview of rheumatoid arthritis and described its incidence throughout the world as well as diagnostic criteria and treatment modalities. Rheumatoid arthritis is a fairly common disease with a myriad of complications. However, we have chosen rheumatoid neuropathy because it is a neurological complication that can be severely debilitating in the long run if early diagnosis and treatment are not provided. In addition, we have compiled several studies that various researchers have conducted to shed light on the occurrence of this complication globally. Even though rheumatoid neuropathy is a common complication, in-depth research is not available because the majority of the cases are subclinical and present late. Therefore, when feasible, timely nerve conduction studies should be conducted in patients with rheumatoid arthritis. This complication can be recognized in its latent stages and treatment can be provided as soon as possible. Ongoing research on this subject seems promising, and we hope that shortly, newer and more effective diagnostic and treatment modalities can be introduced to improve the quality of life of patients.
